# Metformin-induced changes of the gut microbiota in healthy young men: results of a non-blinded, one-armed intervention study

**DOI:** 10.1007/s00125-019-4848-7

**Published:** 2019-03-23

**Authors:** Thomas Bryrup, Cæcilie W. Thomsen, Timo Kern, Kristine H. Allin, Ivan Brandslund, Niklas R. Jørgensen, Henrik Vestergaard, Torben Hansen, Tue H. Hansen, Oluf Pedersen, Trine Nielsen

**Affiliations:** 1 Novo Nordisk Foundation Center for Basic Metabolic Research, Faculty of Health and Medical Sciences, University of Copenhagen, Blegdamsvej 3B, DK-2200 Copenhagen, Denmark; 2 Center for Clinical Research and Prevention, Bispebjerg and Frederiksberg Hospital, The Capital Region, Copenhagen, Denmark; 3 Department of Biochemistry and Immunology, Lillebaelt Hospital, Vejle, Denmark; 4 Institute of Regional Health Research, University of Southern Denmark (SDU), Odense C, Denmark; 5 Dept of Clinical Biochemistry, Rigshospitalet, Copenhagen, Denmark; 6 Odense Patient data Explorative Network (OPEN), Odense University Hospital/Institute of Clinical Research, University of Southern Denmark, Odense, Denmark; 7 Steno Diabetes Center Copenhagen, Gentofte, Denmark; 8 Faculty of Health Sciences, University of Southern Denmark, Odense, Denmark; 9 Department of Cardiology and Endocrinology, Slagelse Hospital, Slagelse, Denmark

**Keywords:** Drug therapy, Gut microbiota, Intervention, Metformin, Microbiome, Microbiota, Type 2 diabetes

## Abstract

**Aims/hypothesis:**

Individuals with type 2 diabetes have an altered bacterial composition of their gut microbiota compared with non-diabetic individuals. However, these alterations may be confounded by medication, notably the blood-glucose-lowering biguanide, metformin. We undertook a clinical trial in healthy and previously drug-free men with the primary aim of investigating metformin-induced compositional changes in the non-diabetic state. A secondary aim was to examine whether the pre-treatment gut microbiota was related to gastrointestinal adverse effects during metformin treatment.

**Methods:**

Twenty-seven healthy young Danish men were included in an 18-week one-armed crossover trial consisting of a pre-intervention period, an intervention period and a post-intervention period, each period lasting 6 weeks. Inclusion criteria were men of age 18–35 years, BMI between 18.5 kg/m^2^ and 27.5 kg/m^2^, HbA_1c_ < 39 mmol/mol (5.7%) and plasma creatinine within the normal range. No prescribed medication, including antibiotics, for 2 months prior to recruitment were allowed and no previous gastrointestinal surgery, discounting appendectomy or chronic illness requiring medical treatment. During the intervention the participants were given metformin up to 1 g twice daily. Participants were examined five times in the fasting state with blood sampling and recording of gastrointestinal symptoms. Examinations took place at Frederiksberg Hospital, Denmark before and after the pre-intervention period, halfway through and immediately after the end of intervention and after the wash-out period. Faecal samples were collected at nine evenly distributed time points, and bacterial DNA was extracted and subjected to 16S rRNA gene amplicon sequencing in order to evaluate gut microbiota composition. Subjective gastrointestinal symptoms were reported at each visit.

**Results:**

Data from participants who completed visit 1 (*n*=23) are included in analyses. For the primary outcome the relative abundance of 11 bacterial genera significantly changed during the intervention but returned to baseline levels after treatment cessation. In line with previous reports, we observed a reduced abundance of *Intestinibacter* spp. and *Clostridium* spp., as well as an increased abundance of *Escherichia/Shigella* spp. and *Bilophila wadsworthia*. The relative abundance at baseline of 12 bacterial genera predicted self-reported gastrointestinal adverse effects.

**Conclusions/interpretation:**

Intake of metformin changes the gut microbiota composition in normoglycaemic young men. The microbiota changes induced by metformin extend and validate previous reports in individuals with type 2 diabetes. Secondary analyses suggest that pre-treatment gut microbiota composition may be a determinant for development of gastrointestinal adverse effects following metformin intake. These results require further investigation and replication in larger prospective studies.

**Trial registration:**

Clinicaltrialsregister.eu 2015-000199-86 and ClinicalTrials.gov NCT02546050

**Funding:**

This project was funded by Danish Diabetes Association and The Novo Nordisk Foundation

**Electronic supplementary material:**

The online version of this article (10.1007/s00125-019-4848-7) contains peer-reviewed but unedited supplementary material, which is available to authorised users.

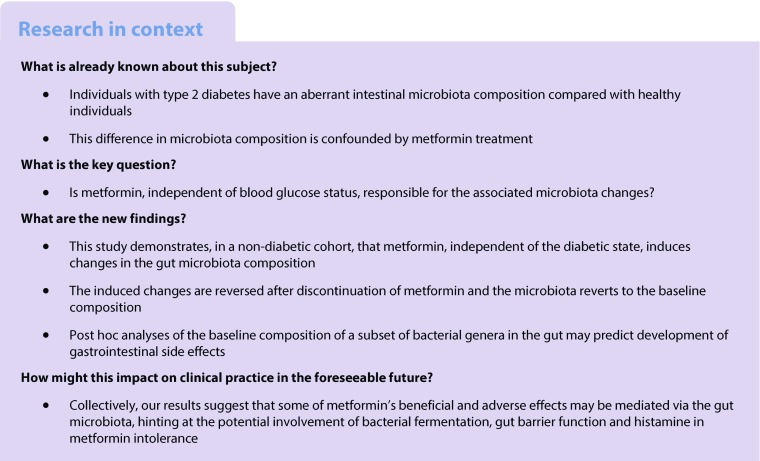



## Introduction

For decades, the biguanide, metformin, has been the first-line oral glucose-lowering drug of choice when treating type 2 diabetes. The antihyperglycaemic effects of metformin include suppression of hepatic gluconeogenesis and increased glucose uptake in skeletal muscle tissue mediated by activation of adenosine monophosphate-activated protein kinase (AMPK) [1–3]. Some studies also suggest the involvement of non-AMPK-mediated suppression of hepatic gluconeogenesis [4, 5]. However, evidence suggests that the gastrointestinal tract and the microorganisms that reside within it are partly involved in mediating both beneficial and adverse effects of metformin. Intraluminal metformin concentration in the gastrointestinal tract is up to 300 times higher than in plasma [6, 7], and metformin treatment decreases intestinal glucose absorption [8]. Furthermore, AMPK activation in duodenal epithelium lowers the plasma glucose concentration in rats [9]. Individuals with type 2 diabetes have an altered composition of the gut microbiota [10–14], and part of the aberration of the microbiota is linked to metformin treatment [14–18]. The gut microbiota is involved in intestinal bile acid metabolism and short-chain fatty acid production, which could explain some of the glucose-lowering effect of metformin, through effects on incretin secretion, hepatic glucose homeostasis and beta cell function [19, 20]. In support of this, data from 22 patients with type 2 diabetes, treated with metformin for 3 days, showed a *Bacteroides fragilis*-linked increase in the intestinal concentration of glycoursodeoxycholic bile acid, which in rodent studies has been associated with improvement of hyperglycaemia [15]. Furthermore, the faecal concentration of the short-chain fatty acid propionate was increased in 15 metformin users compared with nine non-users [21]. Similarly, during a 4 month intervention, faecal butyrate and propionate concentrations were increased significantly in men treated with metformin compared with placebo [18], demonstrating an effect of metformin on fermentative metabolites involved in regulating human metabolism. A hyperglycaemia-independent effect of metformin on the induced perturbation of the gut microbiota has recently been shown after 1 week of metformin intake in 18 healthy individuals [16], and elucidation hereon could further our understanding of the link between type 2 diabetes and the gut microbiota.

The most common side effect of metformin treatment is gastrointestinal discomfort, including nausea, bloating, flatulence and diarrhoea [22, 23]. The mechanisms responsible for these adverse effects are poorly understood but a role of the gut microbiota as a potential mediator has been proposed [14]. Whether the side effects commonly associated with metformin treatment arise from changes in the gut microbiota needs further exploration.

The primary objective of the present intervention in young, healthy men was to investigate compositional changes of the gut microbiota following metformin intake, independent of the physiological changes induced by the diabetic state. In post hoc analyses, we aimed to explore whether the pre-intervention gut microbiota profile was related to self-reported gastrointestinal discomfort following metformin intervention.

## Methods

### Experimental design and study participants

**Fig. 1:**
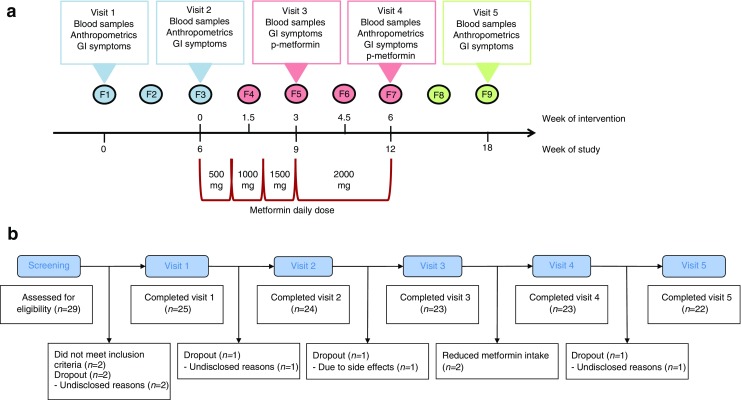
Study design and flow chart. (**a**) One-armed crossover design with five visits and a total of nine faecal samplings (F1–F9). Metformin intervention was initiated after visit 2, with a gradual increase in dose over 3 weeks, from 500 mg to 2000 mg, to minimise adverse effects. Blood samples were drawn at all five visits. Self-reported gastrointestinal symptoms were evaluated at all visits using a VAS. Anthropometrics measurements were taken every 6 weeks and plasma metformin was measured twice during the intervention period to assess compliance. (**b**) Flowchart of study. Twenty-nine men underwent screening. Two participants were ineligible for inclusion. Twenty-seven were included in the trial. Three participants dropped out during the run-in period: two dropped out immediately after the screening visit and another dropped out immediately after the first visit, for undisclosed reasons. One participant dropped out during the intervention due to severe gastrointestinal discomfort. One participant dropped out during the post-intervention follow-up, for undisclosed reasons. Twenty-three completed the intervention period, 22 participants completed the follow-up and 25 were included in the analyses. Two participants reduced metformin intake because of side effects. GI, gastrointestinal; p-metformin, plasma metformin

The study was designed as a non-blinded, non-randomised, one-armed crossover study. Participants were studied for 18 weeks, divided into a run-in period, an intervention period and a wash-out period, each lasting 6 weeks. During the trial period (July 2015 to February 2016), participants were examined at five visits (Fig. 1a) at Frederiksberg Hospital, Denmark.

Participants were eligible for enrolment if they were men, age 18–35 years, had a BMI between 18.5 kg/m^2^ and 27.5 kg/m^2^, HbA_1c_ < 39 mmol/mol (5.7%) and plasma creatinine within the normal range. Inclusion criteria included no prescribed medication, including antibiotics, for 2 months prior to recruitment, no previous gastrointestinal surgery discounting appendectomy or chronic illness requiring medical treatment. Participants were instructed not to change dietary habits and lifestyle during the study period.

The intervention was initiated after the second visit, 6 weeks after inclusion. All study participants were instructed to take metformin according to a fixed schedule: 500 mg once daily for the first week, 500 mg twice daily for the second week, 1000 mg+ 500 mg daily for the third week and 1000 mg+ 1000 mg daily for the remaining 3 weeks of the intervention period.

The study was designed for 25 men starting the intervention, expecting a 20% dropout.

The trial was conducted according to the International Conference on Harmonization’s Good Clinical Practice guidelines including the Declaration of Helsinki II. The study was approved by the Ethical Committees of the Capital Region of Denmark (protocol ID: H-7-2014-012) and was registered at www.clinicaltrialsregister.eu (no. 2015-000199-86) and at ClinicalTrials.gov (NCT02546050). All participants gave written informed consent before taking part in the study. No changes to methods were made after trial commencement. The trial was concluded when all the participants had finalised their last visit. No serious adverse events were reported during the trial.

### Clinical data

Participants underwent clinical examination five times during the trial: at baseline (visit 1), at the end of the run-in (week 6; visit 2), 3 weeks into the intervention period (week 9; visit 3), at the end of the intervention period (week 12; visit 4) and at the end of the wash-out period (week 18; visit 5). Gastrointestinal symptoms (overall abdominal discomfort and degree of abdominal pain, bloating, constipation, diarrhoea, flatulence, metal taste, nausea and stool consistency satisfaction) were evaluated at all five visits using a digital visual analogue scale (VAS) and recorded as an integer between 0 and 100.

At visits 1, 2, 4 and 5, respectively, participants were examined with height, weight, hip and waist circumference, BP and bioimpedance. Detailed information on anthropometrics can be found in electronic supplementary material (ESM) Methods. A questionnaire on quality of life, physical activity and health status was completed at baseline.

Blood samples were collected at all visits after participants had fasted for 10 h overnight. Alanine aminotransferase and cobalamin were analysed using an enzymatic slide test, and creatinine, glucose, cholesterol, HDL cholesterol, and triglyceride were measured using a colorimetric slide test. LDL cholesterol was calculated using the Friedewald formula [24]. HbA_1c_ was measured using liquid chromatography and insulin was measured using immunoassay. Leukocytes and differential white blood cell count were measured using flow cytometry. Plasma metformin concentration was measured using high performance liquid chromatography followed by tandem mass spectrometry to ensure compliance. Detailed information on biochemical measurements is presented in the ESM Methods. HOMA of insulin resistance and beta cell function were calculated from fasting plasma concentrations of glucose and insulin according to Matthews et al [25].

Participants were divided into two groups based on change in reported overall abdominal discomfort. An increase in VAS score at visit 3 exceeding 2 × SEM at baseline was considered significant and used as a cut-off. One participant who dropped out before visit 3 due to severe gastrointestinal discomfort was counted among those who developed gastrointestinal adverse effects in spite of missing VAS data. By this definition, 10 of 23 participants experienced an increase in overall abdominal discomfort.

### Faecal samples and data processing

Participants were instructed to deliver nine faecal samples throughout the study period. Samples were collected on the day of examination if possible and immediately frozen either at the study site or at home, in which case samples were transferred to the laboratory on dry ice. Samples were stored at −80°C until DNA extraction.

A total of 206 faecal samples were collected but two samples did not contain sufficient material for DNA extraction. Genomic DNA was isolated, followed by PCR amplification of the V4 hypervariable region of the bacterial 16S rRNA gene and sequencing on an Illumina MiSeq platform generating a total of 9,466,021 (mean 43,622 [SD 18,700]) paired-end (250 bp) reads. See ESM Methods for detailed information. Twelve samples that failed during the first run were resequenced. Processing of raw sequencing data was performed using the dada2 (v1.4.0) R package [26]. Following inspection of quality profiles, denoising, merging of reads and removal of chimeric sequences, a total of 7,214,117 reads (mean 34,702 [SD 12,889]; minimum 10,736) in 1764 unique amplicon sequence variants (ASVs) were available for downstream analyses. Taxonomical assignment of ASVs from kingdom to species was performed against the Silva v128 database, using the dada2 implementation of the naive Bayesian RDP classifier [27].

#### Statistical analyses

The primary outcome was compositional change in abundance of ASVs agglomerated at the taxonomic level of genus. Pre-specified secondary outcomes were as follows: compositional change in abundance of non-agglomerated ASVs; change in intra-individual diversity quantified by the number of observed ASVs (richness), Shannon’s entropy and Pielou’s evenness (Shannon’s entropy/log_*e*_[richness]); change in community structure and membership quantified by Canberra and Jaccard distances, respectively, and change in anthropometric and biochemical traits. Post hoc analyses included random forest (RF) classification of individuals developing gastrointestinal discomfort and testing for bacterial genera and ASVs responding differently to metformin in these participants, compared with those who did not develop gastrointestinal side effects. Data from 24 participants examined at least once during the intervention period were included in these subanalyses. Data from participants not following protocol (e.g. reduced metformin intake, *n* = 2) were omitted from analyses from the time they deviated from protocol. In total, 25 participants completed visit 1 and were included in the statistical analyses, but one participant dropped out before initialisation of the intervention.

ASV abundances were agglomerated at genus level using the phyloseq R package, normalised using total sum scaling, and log transformed following addition of a pseudocount corresponding to the lowest non-zero normalised abundance of each taxon. Intervention effect was modelled by a one-way repeated measures ANOVA using mixed linear regression as implemented in the lme4 R package. Models were fitted using restricted maximum likelihood using samples from all time points. The abundance of each genus at each time point during the metformin intervention was compared by a post hoc *t* test with the mean abundance averaged over the three samples collected during the pre-intervention period. Genera never present in >80% of participants were excluded from analyses. Intervention effect on non-agglomerated ASV abundances, bacterial richness, Shannon’s entropy and Pielou’s evenness was assessed using the same approach considering a two-tailed *p* value <0.05 as significant. Correction for false discovery rate was done for all genera/ASVs across all time points by the Benjamini–Hochberg method, applying a false discovery rate of 10% for significance.

Intervention effect on community structure and community membership was assessed by permutational multivariate ANOVA (PERMANOVA; vegan R Package v2.4.6) of Canberra and Jaccard distances, contrasting each intervention and post-intervention time point to the average across the three pre-intervention time points. Principal coordinate ordination was performed using the capscale function of the vegan package, specifying an unconstrained model.

RF classification was used to identify bacterial genera that at baseline were discriminative for the development of gastrointestinal adverse effects during the intervention. RF models were trained on total sum scaled abundances of 124 bacterial genera using the caret (v6.0.79) and randomForest (v4.6.12) R packages with 25 iterations of bootstrap resampling. Down-sampling during resampling was applied to address class imbalance. Performance across resamples was evaluated by the area under the receiver operating characteristic (ROC) curve and discriminant genera were ranked by the mean decrease in accuracy. Using the optimal variable settings from the naive RF classifier (mtry = 2), we applied the Boruta (v5.2.0) R package for feature selection of all-relevant discriminant genera identified across 200 repetitions using different random seeds. Selected genera were subsequently used to build an optimal RF classifier, as described above.

We used mixed linear regression to identify bacterial genera and ASVs responding differently to the metformin intervention in participants who developed gastrointestinal side effects compared with those who did not. A response profile model was specified with a main effect of time (categorical) and a time × group interaction as fixed effects and a random intercept for each participant, thereby testing the difference between groups at all time points during the intervention. Data were corrected for multiplicity as described above.

Change in clinical and biochemical traits was assessed using linear mixed model regression ANOVA as outlined above. Logarithmic transformation of the dependent variable was applied if deemed appropriate upon inspection of residual plots and normal probability plots. For VAS data, a constant of 1 was added prior to transformation. Difference in plasma metformin between visit 3 and 4 were tested with a Wilcoxon signed-rank test.

All statistical analyses were made using R v3.4.2 (https://www.R-project.org/).

## Results

### Study population characteristics

Twenty-nine men were assessed for eligibility, two participants did not meet inclusion criteria. Two participants dropped out before visit 1 and were excluded from analyses. One participant discontinued after visit 1. Twenty-four participants started the intervention with metformin and 25 participants with relevant data were included in analyses (Fig. 1b).

Study population characteristics throughout the trial are presented in Table 1. Briefly, participants had a mean age of 26 years (SD 3.4), were lean with median BMI of 22.9 kg/m^2^ (SD 2.1), had a fat percentage of 14.0% (SD 3.3) and were normoglycaemic with fasting plasma glucose 5.3 mmol/l (SD 0.4) and HbA_1c_ 33.4 mmol/mol (5.2%) (SD 2.9 and 0.26, respectively) at baseline.

**Table 1:** Characteristics of the study population

Characteristic	Visit 1 (0 mg metformin)	Visit 2 (0 mg metformin)	Visit 3 (1500 mg metformin)	Visit 4 (2000 mg metformin)	Visit 5 (0 mg metformin)
*n*	25	24	23	23	22
Age, years	26 ± 3.4				
BMI, kg/m^2^	22.9 ± 2.1	22.8 ± 2.0		22.8 ± 1.9	22.8 ± 2.0
Body fat, %	14.0 ± 3.3	14.3 ± 3.0		14.4 ± 3.0	14.6 ± 3.3**
Waist/hip ratio	0.88 ± 0.03	0.88 ± 0.04		0.87 ± 0.04	0.90 ± 0.06**
Systolic BP, mmHg	123 ± 11.6	125 ± 9.0		122 ± 7.8	124 ± 7.1
Diastolic BP, mmHg	72 ± 5.5	71 ± 6.8		71 ± 8.9	72 ± 8.4
Fasting plasma glucose, mmol/l	5.3 ± 0.4	5.2 ± 0.3	5.2 ± 0.3	5.2 ± 0.3	5.4 ± 0.5
Fasting serum insulin, pmol/l	42 (32–51)	35 (32–57)	45 (30–57)	35 (25–50)	42 (32–59)
HOMA insulin resistance index	1.61 (1.19–2.11)	1.34 (1.18–2.12)	1.76 (1.11–2.32)	1.32 (0.96–1.92)	1.62 (1.20–2.38)
HOMA beta cell function, %	76 (64–89)	80 (57–106)	87 (65–101)	65 (54–110)	86 (55–105)
HbA_1c_, mmol/mol	33.4 ± 2.9	33.1 ± 3.0	32.4 ± 2.6*	32.4 ± 2.9**	33.3 ± 2.8
HbA_1c_, %	5.2 ± 0.26	5.2 ± 0.28	5.1 ± 0.24*	5.1 ± 0.26**	5.2 ± 0.26
Fasting plasma B_12_ pmol/l	320 (262–408)	327 (264–443)	298 (257–459)	281 (251–389)**	392 (283–437)*
Fasting blood total leucocytes, ×10^9^/l	5.9 ± 1.8	5.7 ± 1.4	5.8 ± 1.3	5.8 ± 1.0	6.0 ± 1.4
Fasting plasma total cholesterol, mmol/l	4.31 ± 0.8	4.41 ± 0.82	4.38 ± 0.90	4.15 ± 0.83**	4.55 ± 0.84*
Fasting plasma metformin, nmol/l			399 (246–507)	449 (292–660)	

Data are displayed as mean ± SD or median (interquartile range)

Visits 3, 4 and 5, respectively, were tested vs a combined baseline averaged across visit 1 and 2 using mixed linear regression. Difference in plasma metformin between visit 3 and 4 was tested with a Wilcoxon signed-rank test

*p < 0.05 and **p < 0.01 vs baseline

Linear mixed effect modelling revealed a significant increase in body fat percentage (0.98%, *p* = 0.002) and waist/hip ratio (0.041, *p* = 0.002) at visit 5 compared with a combined baseline value of visit 1 and 2. A significant decrease in HbA_1c_ was detected at visit 3 (−1.15 mmol/mol [−0.11%], *p* = 0.03) and visit 4 (−1.67 mmol/mol [−0.15%], *p* = 0.001). There was also a decrease in plasma B_12_ (−17.20%, *p* = 0.001) and plasma cholesterol (−0.49 mmol/l, *p* = 0.007) levels at visit 4 and a counter response with significantly elevated levels of these two variables at visit 5 (13.87%, *p* = 0.03 and 0.40 mmol/l, *p* = 0.03, respectively).

### Metformin changes gut microbiota composition in healthy adults

**Fig. 2:**
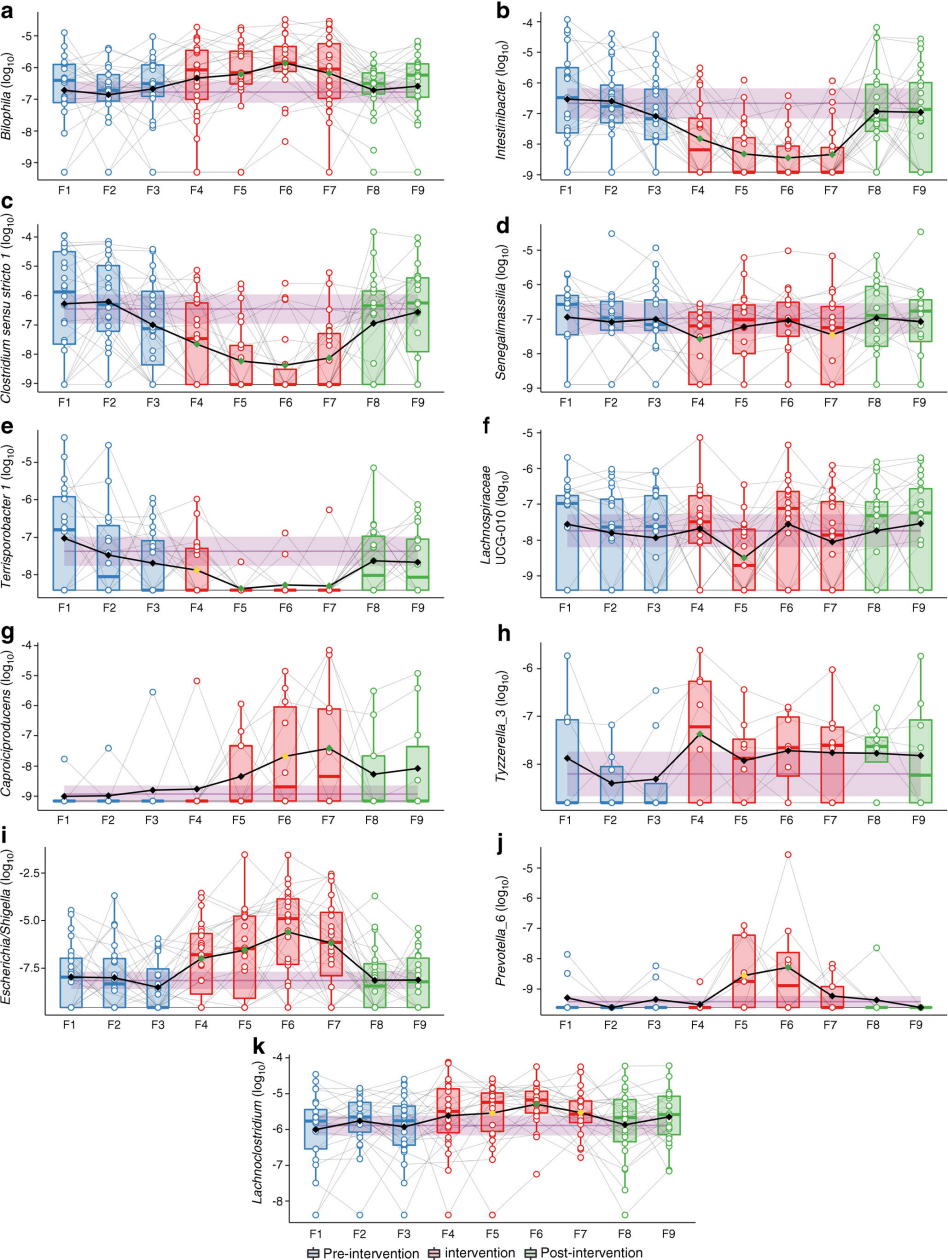
Metformin-responsive bacterial genera exhibiting a change in relative abundance during the metformin intervention. Boxes represent interquartile range (IQR), with the inner horizontal line representing the median. Whiskers represent values within 1.5 × IQR of the first and third quartiles. Circles represent individual samples with lines connecting samples from the same individual. The purple band represents the pre-intervention mean and 95% confidence limits averaged across the three pre-intervention time points. Diamonds and connecting lines represent mean values, with yellow and green diamonds, respectively, representing nominal (*p* < 0.05) and false discovery rate adjusted (*q* < 0.05) significant differences from the averaged pre-intervention mean. The relative abundance at each time point during the intervention was compared with the averaged pre-intervention mean by linear mixed model regression ANOVA. Only genera with a significant change at least at one time point following correction for false discovery rate are presented

The primary outcome was compositional changes in the gut microbiota at genus level. The abundance of five bacterial genera was significantly decreased and the abundance of six was significantly increased during the metformin intervention at a false discovery rate of 10%, at least once during the intervention period (Fig. 2, ESM Fig. 1 and ESM Table 1). All returned to baseline levels after treatment cessation. Of the genera decreasing in abundance, *Intestinibacter*, *Clostridium* (*Clostridium* sensu stricto *1*) and *Terrisporobacter* decreased immediately after treatment initiation and remained low throughout the intervention period. The abundance of *Senegalimassilia* decreased immediately after treatment initiation but reverted to baseline at subsequent time points. The abundance of an unclassified *Lachnospiraceae* (UCG-010) genus was unaffected during the first 10 days (F4) (Fig. 1a) of metformin treatment, decreased 3 weeks (F5) into the intervention after the dose was increased to 1500 mg daily and then subsequently reverted to baseline levels. Among the bacterial genera that increased in abundance, *Escherichia*/*Shigella* increased immediately after treatment initiation (F4) and remained significantly increased throughout the intervention period. Likewise, *Bilophila* increased throughout the intervention, but this increase was not evident until week 3 (F5) in the intervention. The increase in the abundance of *Lachnoclostridium* was nominally significant 3 weeks (F5) into the intervention period and this increase became significant compared with baseline at 4.5 weeks (F6), after which time this genus began to revert to baseline levels (remaining increased compared with baseline at nominal significance). *Caproiciproducens* showed a nominally significant increase 4.5 weeks after treatment initiation (F6) and became significantly increased after 6 weeks of metformin treatment (F7). *Tyzzerella* (*Tyzzerella*_3) was increased immediately after treatment but reverted to baseline at the subsequent time points. A *Prevotella* genus (*Prevotella*_6) increased in abundance with nominal significance after 3 weeks and significantly at 4.5 weeks of treatment (F6) but reverted to baseline before treatment cessation.

We identified five ASVs that decreased during the intervention, one of which was assigned to *Clostridium* (ASV_156), one to *Intestinibacter bartlettii* (ASV_128), one to *Lachnospiraceae* (ASV_247), another to *Terrisporobacter* (ASV_239) and one to *Peptostreptococcaceae* (ASV_80). Twelve ASVs increased, among which four belonged to the family *Lachnospiraceae*, including one assigned to *Lachnoclostridium* (ASV_178), one was assigned to *Escherichia/Shigella* (ASV_40), another to *Alistipes finegoldii* (ASV_94) and one to *Bilophila wadsworthia* (ASV_110) (ESM Fig. 1, ESM Table 2).

### No uniform effect of metformin on community structure or diversity

We did not see any effect of metformin treatment on gut microbiota richness (*p* = 0.60–0.80), evenness (*p* = 0.06–0.99) or diversity (*p* = 0.08–0.90) at any time point during the intervention (ESM Fig. 2). Conversely, we found a significant change in community structure (Canberra distances) at genus level (*R*^2^ = 0.31–0.69%; *p* = 0.001–0.047) at all time points during the intervention. The change in community membership (Jaccard distances) was less pronounced and only significant after 4.5 weeks (*R*^2^ = 0.62%; *p* = 0.003) and 6 weeks (*R*^2^ = 0.43%; *p* = 0.03) of metformin intervention. However, principal coordinate ordination analysis demonstrated that the change during the intervention period did not reflect a uniform shift, but individual changes in community structure and membership (ESM Fig. 3).

### Pre-intervention gut microbiota associated with gastrointestinal side effects

**Fig. 3:**
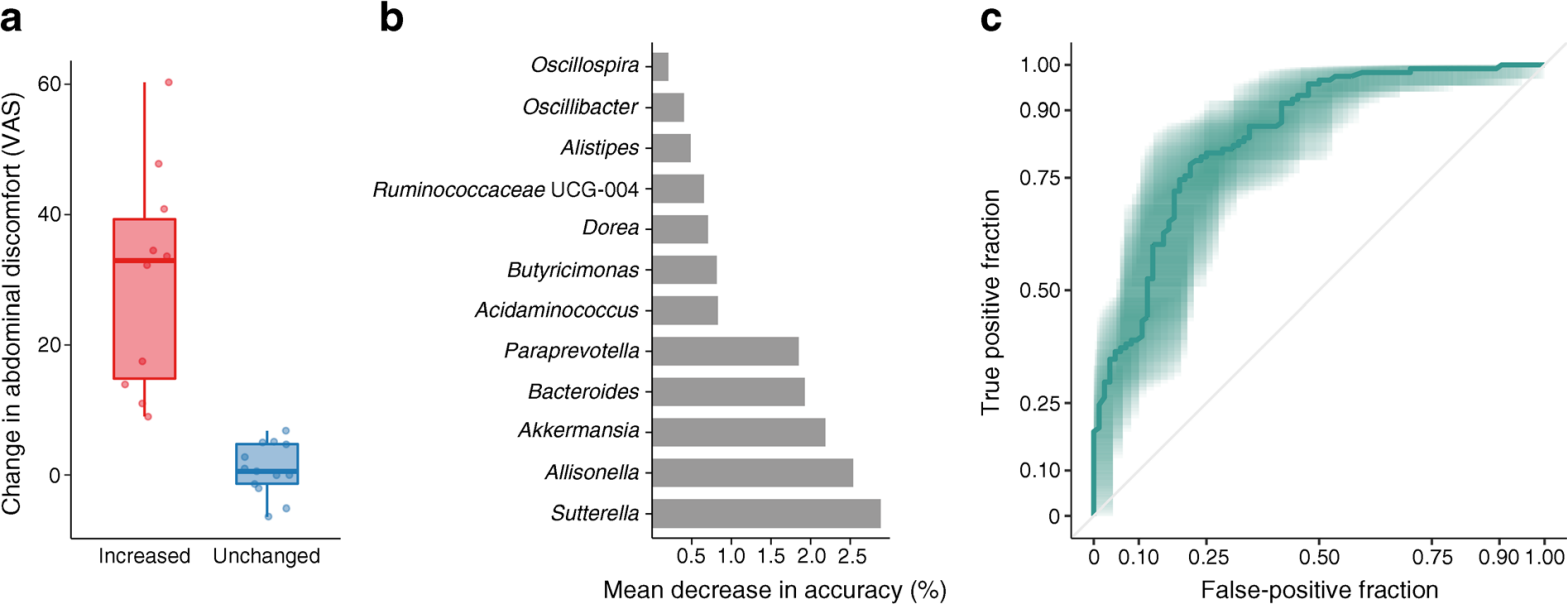
Bacterial genera discriminant for development of gastrointestinal side effects. (**a**) Participants were divided into two groups based on change in overall self-reported gastrointestinal side effects measured on a VAS from baseline to visit 3 (3 weeks into the metformin intervention). Boxes represent interquartile range (IQR), with the inner horizontal line representing the median, whiskers representing values within 1.5 × IQR of the first and third quartiles and circles representing individual samples. (**b**) Importance of bacterial genera identified by Boruta feature selection as being discriminant at baseline for development of gastrointestinal discomfort during metformin intervention. Genera are ordered by mean decrease in accuracy from an RF model based on baseline abundances of the 12 discriminant genera fitted using bootstrap resampling. (**c**) ROC curve representing the ability of the RF model to discriminate between participants who develop gastrointestinal discomfort and those who do not. The shaded area represents the 95% CI; AUC = 0.9

Self-reported measures of gastrointestinal discomfort are presented in Table 2. Overall, we saw an increase in gastrointestinal discomfort, represented by an increase in the severity of abdominal pain (404% [95% CI 91, 1225]; *p* = 0.0011), nausea (392% [95% CI 78, 1258]; *p* = 0.002), bloating (283% [95% CI 31, 1007]; *p* = 0.01) and diarrhoea (261% [95% CI 23, 959]; *p* = 0.02) and an increase in overall abdominal discomfort (67% [95% CI 216, 1800]; *p* < 0.001) at the first visit (3 weeks) after initiation of the intervention. All side effects diminished towards the end of the intervention period. There was, however, substantial inter-individual variation in gastrointestinal adverse effects, with some individuals developing severe discomfort and others experiencing only mild side effects or none at all. We dichotomised the study group based on the change in overall abdominal discomfort from baseline to the first visit during the intervention: ten participants who developed abdominal side effects and 13 who did not (Fig. 3a). When building an RF classifier on baseline abundances of 124 unselected bacterial genera, we were able to moderately discriminate between participants who developed gastrointestinal side effects and those who did not, with an area under the ROC curve of 0.70 (95% CI 0.79, 0.60). Repeated feature selection identified 12 bacterial genera having discriminative importance (Fig. 3b), with *Sutterella*, *Allisonella*, *Akkermansia*, *Bacteroides* and *Paraprevotella* as the main discriminant genera. By building an RF classifier based on the baseline abundances of these 12 genera, we were able to distinguish participants who developed gastrointestinal side effects from those who did not, with a ROC AUC of 0.90 (95% CI 0.94, 0.87) (Fig. 3c). No bacterial genera or ASVs changed differently in the two groups during the metformin treatment.

**Table 2:** Self-reported gastrointestinal adverse effects throughout the trial

Adverse effect	Visit 1	Visit 2	Visit 3	Visit 4	Visit 5
*n*	25	24	23	23	22
Overall abdominal discomfort	6.5 (1.6–15.2)	4 (0–9.6)	13.5 (6.5–27.2)***	7.5 (1–17.5)	0.3 (0–5.9)**
Abdominal pain	0.9 (0–5)	1 (0–8.5)	6.9 (0.6–20)**	0.6 (0–6.5)	0 (0–5.8)
Bloating	3.6 (0–8.4)	2.3 (0.5–6)	5.7 (0.1–26.4)*	6.1 (1.2–18.4)*	0 (0–3.8)
Constipation	0 (0–2.4)	1.5 (0–9.4)	0.6 (0–7.6)	0.5 (0–2.9)	0 (0–3.2)
Diarrhoea	0.1 (0–3.3)	0 (0–4)	0.9 (0–17.6)*	0 (0–15.5)	0 (0–0.6)
Flatulence	6.7 (3–16.7)	6.1 (0.9–14)	11.1 (2.2–27.8)	15.2 (1.6–21.8)	7.7 (0–14.2)
Metallic taste	0.1 (0–1.9)	0 (0–5.9)	0.2 (0–2.4)	0 (0–1.9)	0 (0–0.8)
Nausea	1 (0–3.5)	1.6 (0–7.5)	4.5 (0.1–19.2)**	1 (0–7.9)	0 (0–0.4)
Stool consistency satisfaction	12.8 (0.7–32.3)	9.5 (2.1–25)	24.7 (3–32)	11.3 (3.4–28)	6.4 (0–12.5)

Data are displayed as median (interquartile range)

Symptoms were evaluated using a digital VAS recording severity as an integer from 0 (none at all) to 100 (worst ever). Visits 3, 4 and 5, respectively, were tested vs a combined baseline averaged across visit 1 and 2 using mixed linear regression

*p < 0.05, **p < 0.01 and ***p < 0.001 vs baseline

## Discussion

We show that metformin intervention has an impact on the composition of the human gut microbiota at genus and ASV levels in healthy young men, and these changes are reversed after discontinuation of metformin. In post hoc, secondary aim analyses, we demonstrate that the pre-treatment composition of a subset of bacterial genera may predict risk of development of gastrointestinal adverse effects to metformin.

Until now, several studies have recognised that metformin treatment associates with a structural change of the gut microbial community. A double-blinded randomised study [18] of 40 treatment-naive patients with type 2 diabetes given placebo or metformin for 4 months showed an increase in abundance of *Escherichia* spp. and *Bilophila wadsworthia* along with a decrease in *Intestinibacter* spp. and *Clostridium* spp. Similarly, using shotgun sequencing-based metagenomic analyses we previously reported an increase of *Escherichia* spp. and a reduced abundance of *Intestinibacter* spp. in metformin-treated type 2 diabetes patients [14]. In a recent study of 18 healthy participants given 850 mg metformin twice daily for 1 week an increase of *Escherichia/Shigella* was also reported [16]. Our analyses of healthy normoglycaemic individuals similarly showed a reduced abundance of *Intestinibacter* spp. and *Clostridium* spp., as well as an increased abundance of the genus *Escherichia/Shigella* and *B. wadsworthia* in response to metformin treatment. Collectively, the findings substantiate that the effect of metformin is independent of the dysbiosis induced by diabetes. Additionally, we identified seven genera changing in abundance during the metformin intervention, further demonstrating that metformin treatment has a profound impact on the gut microbiota.

Consistent correlations between alterations of gut microbiota composition and metformin intake, along with evidence that the intended effect of metformin is possibly co-mediated by the gut, suggest that alterations of the gut microbiota contribute to the therapeutic effect of metformin. However, long-term prospective studies of diabetic patients treated with metformin could determine causality. Identifying such a causality could be the first step in performing bacteria-based interventions in type 2 diabetes patients.

Adverse gastrointestinal effects are the primary cause of failing metformin compliance. In a recent study of 18 healthy young men exposed to metformin for 1 week, an association between increased abundance of *Escherichia*/*Shigella* spp. prior to the intervention and later development of gastrointestinal side effects was reported [16]. However, no formal statistical evidence was provided to substantiate this finding. In the present study, we aimed to identify genera associated with development of gastrointestinal adverse effects. The composition of 12 bacterial genera of the pre-intervention gut microbiota was identified as a possible predictor of self-reported gastrointestinal adverse effects during metformin treatment. Among these, the highly abundant genera *Sutterella*, *Akkermansia* and *Allisonella* had the greatest predictive value [28]. Interestingly, *Sutterella* spp. have been associated with infections of the gastrointestinal tract, inflammatory bowel disease [29] and autism [30].

*Akkermansia muciniphila* is one of the most abundant bacterial species of the human gut microbiota and specialises in mucin degradation. Using the glycosylated proteins of the epithelial mucus layer as its major source of carbon and nitrogen in fermentative processes producing the short-chain fatty acids acetate and propionate, *A. muciniphila* strengthens the integrity of the intestinal epithelium and regulates the gut barrier function [31]. In studies in animals [32] and humans [17, 33], metformin treatment was associated with increased abundance of *A. muciniphila* and the bacterium has been linked to improved glycaemic control [34]. We did not identify *A. muciniphila* among the metformin-responsive bacteria in the present study, perhaps because metformin treatment in individuals with diabetes resets a perturbation in *A. muciniphila* abundance caused by the disease; an imbalance that is not present in healthy individuals.

The genus *Allisonella*, and the only known species *A. histaminiformans*, produces histamine from histidine [35]. Histamine is a potent vasoactive agent causing vasodilatation and increased vascular permeability, as well as a potent inducer of mucus secretion. Histamine is capable of inducing stomach ache, cramps, meteorism and diarrhoea, which are all known gastrointestinal side effects of metformin treatment [36]. Interestingly, in vitro studies have shown that metformin inhibits degradation of histamine by diamine oxidase at concentrations achieved in the intestine after therapeutic doses [37]. Whether metformin affects histamine production by the gut microbiota, how that may interact with effects on host capacity for histamine degradation to induce gastrointestinal intolerance and how the intolerance might be prevented or treated require testing in future studies. Yet, our findings of gastrointestinal side effects associated with pre-treatment bacterial composition must be interpreted with caution as our dataset is limited by its size and lack of validation in an independent cohort. Furthermore, there is a risk of overestimation due to small sample size and the large number of features in the model. Despite this major limitation, our data generate a hypothesis for future studies aiming to limit gastrointestinal side effects in patients introduced to metformin by modulating intestinal bacterial composition before medication is given.

A strength of the study is that multiple faecal samples were collected prior to the intervention (three samples over 6 weeks), enabling us to account for background fluctuations in gut microbiota composition, thereby improving the reliability of intra-individual modelling. Longitudinal sampling during and after the intervention also demonstrates the dynamics of the gut microbiota in response to the initiation and cessation of treatment. The design is, however, underpowered to test inter-individual effects, which is presumably why we were unable to identify bacterial genera or ASVs responding differently to the metformin intervention in participants who developed or did not develop gastrointestinal side effects. Much larger sample sizes are required to reliably identify interventional effects in between-group analyses [38]. Other limitations include the lack of blinding and inclusion of a placebo control group. Technologically, the study was limited by usage of the 16S rRNA gene amplicon sequencing approach, which is well suited for detecting signals at the genus level but provides limited insight at the species level.

In conclusion, the blood glucose-lowering biguanide metformin changes the composition of the intestinal microbiota independent of the prevailing blood glucose level, showing that the effect is independent of the dysbiosis induced by diabetes. We propose that the pre-treatment composition of a subset of bacterial genera in the gut may predict risk of gastrointestinal side effects, hinting at the potential involvement of bacterial fermentation, gut barrier function and histamine in metformin intolerance.

## Electronic supplementary material

ESM (PDF 1.40 MB)

ESM Tables (XLSX 30 kb)

## Data Availability

Data are available from the authors upon reasonable request.

## Abbreviations

AMPK: Adenosine monophosphate-activated protein kinase
ASV: Amplicon sequence variant
PERMANOVA: Permutational multivariate ANOVA
RF: Random forest
ROC: Receiver operating characteristic
VAS: Visual analogue scale
