# Supplementary material for: Metformin-induced changes of the gut microbiota in healthy young men: results of a non-blinded, one-armed intervention study
Source: Diabetologia. 2019 Mar 23;62(6):1024–35. doi: 10.1007/s00125-019-4848-7 (PMC6509092; doi:10.1007/s00125-019-4848-7)
Supplement: Supplementary file 1 — (PDF 1.40 MB) [file 125_2019_4848_MOESM1_ESM.pdf]

ESM Methods

Clinical examination Height was measured without shoes to the nearest 0.5 cm using a wall mounted stadiometer (MZ10023, ADE, Germany). Weight was measured with participants wearing underwear to the nearest 0.1 kg (WB-110MA, Tanita Corporation of America, IL USA). Hip and waist circumference were recorded as the average of duplicate measurements to the nearest 0.5 cm using a non-expandable measuring tape. Waist circumference was measured midway between the lower rib margin and the iliac crest. Hip circumference was measured as the largest circumference between the waist and the thighs. Blood pressure was measured in the inclined position after a minimum 10 min rest using an automated sphygmanometer (UA-779, A&D medical, Japan) and recorded as the last of triplicate measurements. Body composition was assessed using a bioelectrical impedance analyzer (Tanita Body Composition Analyzer BC-420MA).

Biochemical analyses Plasma concentrations of alanine aminotransferase and cobalamin were analyzed on a Vitros 5,1 FS/5600 (Ortho Clinical Diagnostics, NJ USA) platform, using multipoint enzymatic slide test and immunometric analysis with a CV of 19.7-33.0% and 6.0%, respectively. Creatinine, glucose, cholesterol, HDL cholesterol, and triglyceride were measured on the Vitros 5,1 FS/5600 platform using colorimetric slide test with CV 12.6%, 6.1%, 11.6%, 17.0% and 14.6%, respectively. LDL cholesterol was calculated using the Friedewald formula[1]. HbA_{1c} was measured on a TOSOH G8 (Tosoh Bioscience, CA USA) platform using high performance liquid chromatography with a CV of 7.2%. Insulin concentration in plasma was quantified on a Roche Cobas 411 (Roche Diagnostics GmbH, Germany) platform using an enzyme-linked chemiluminescent immunoassay with a CV of 2.8%. Leukocytes and differential white blood cell count were measured on an Advia platform using coupled flow cytometry and peroxidase methods. Plasma metformin was measured by high performance liquid chromatography followed by tandem mass spectrometry (LC-MS/MS) at visit 3 and 4, respectively, in order to evaluate compliance. In brief, proteins were precipitated with acetonitrile containing the deuterated internal standard, metformin-d6 hydrochloride, and the supernatant diluted by acetonitrile. The analysis was performed on an ACQUITY UPLC I-class system connected to a Xevo TQ-S (Waters Corporation, MA USA) tandem mass spectrometer in electrospray positive ionization mode. Separation was achieved on a XBridgeT BEH Amide 2.5 µm column (Waters Corporation, MA USA) and gradient elution

with i) 100mM ammonium formate pH 3.2 and ii) acetonitrile. The multiple reaction monitoring transitions used for metformin and metformin-d6 were 130.2>71.0 and 136.2>60.0. Calibrators were prepared by spiking drug free serum with metformin to a concentration of 2000 ng/mL.

16S rRNA sequencing Genomic DNA was isolated from approximately 200 mg of frozen stool using the NucleoSpinSoil kit (Macherey-Nagel GmbH & Co. KG, Germany) following the manufacturer's instruction. For the lysis of bacterial cells buffer SL2 + Enhancer buffer SX was used. DNA yield, purity and integrity were assessed using a Qubit 2.0 fluorometer, a NanoDrop 2000 spectrometer (Thermo Fisher Scientific Inc., MA USA) and agarose gel electrophoresis, respectively. 16S rRNA genes were amplified using the primer set 515F (5' AATGATACGGCGACCACCGAGATCTACAC <i5> TATGGTAATTGTGTGCCAGCMGCCGCGGTAA 3') and 806R (5' AAGCAGAAGACGGCATACGAGAT <i7> AGTCAGTCAGCCGGACTACHVGGGTWTCTAAT 3') targeting the V4 hypervariable region [2, 3]. PCR amplification was performed in a total volume of 25 µl, containing 20 ng DNA, 0.2 µM of each primer, and HotMasterMix (5Prime) solution under the following conditions: 3 min at 94°C, followed by 28 cycles of 20s at 94°C, 30s at 55°C and 54s at 72°C on an Eppendorf thermocycler (Eppendorf AG, Germany). The samples were purified with a magnetic-bead based clean-up and size selection kit (Macherey-Nagel GmbH & Co. KG, Germany). A master DNA pool was generated from the purified products in equimolar ratios. The DNA was sequenced on an Illumina MiSeq platform (MiSeq Reagent Kits v2, 500 cycles), generating a total of 9,466,021 (mean = 43622; SD = 18700) paired-end (250 bp) reads.

Processing of raw sequencing data was performed using the *dada2* (v1.4.0) R package [4]. Following inspection of quality profiles, the first 10 nt of all reads were trimmed, with forward and reverse reads truncated at 220 nt and 155 nt, respectively. Reads containing ambiguous nucleotides or more than two expected errors were discarded. Sequencing error rates were estimated based on a random subset of 2×10^6 reads. Reads were dereplicated and denoised sample-wise, followed by merging of forward and reverse reads requiring a minimum overlap of 20 nt, with no mismatch allowed. Chimeric sequences were detected and removed, leaving 7,214,117 reads (mean = 34,702, SD = 12,889; minimum = 10,736) in 1,764 unique amplicon sequence variants (ASV) for downstream analyses. Taxonomical assignment

of ASVs from kingdom to species was performed against the Silva v128 database, using the *dada2* implementation of the naïve Bayesian RDP classifier.

[1] Warnick GR, Knopp RH, Fitzpatrick V, Branson L (1990) Estimating low-density lipoprotein cholesterol by the Friedewald equation is adequate for classifying patients on the basis of nationally recommended cutpoints. Clinical chemistry 36: 15-19
[2] Caporaso JG, Lauber CL, Walters WA, et al. (2012) Ultra-high-throughput microbial community analysis on the Illumina HiSeq and MiSeq platforms. ISME J 6: 1621-1624
[3] Kozich JJ, Westcott SL, Baxter NT, Highlander SK, Schloss PD (2013) Development of a dual-index sequencing strategy and curation pipeline for analyzing amplicon sequence data on the MiSeq Illumina sequencing platform. Appl Environ Microbiol 79: 5112-5120
[4] Callahan BJ, McMurdie PJ, Rosen MJ, Han AW, Johnson AJ, Holmes SP (2016) DADA2: High-resolution sample inference from Illumina amplicon data. Nat Methods 13: 581-583

ESM Figures

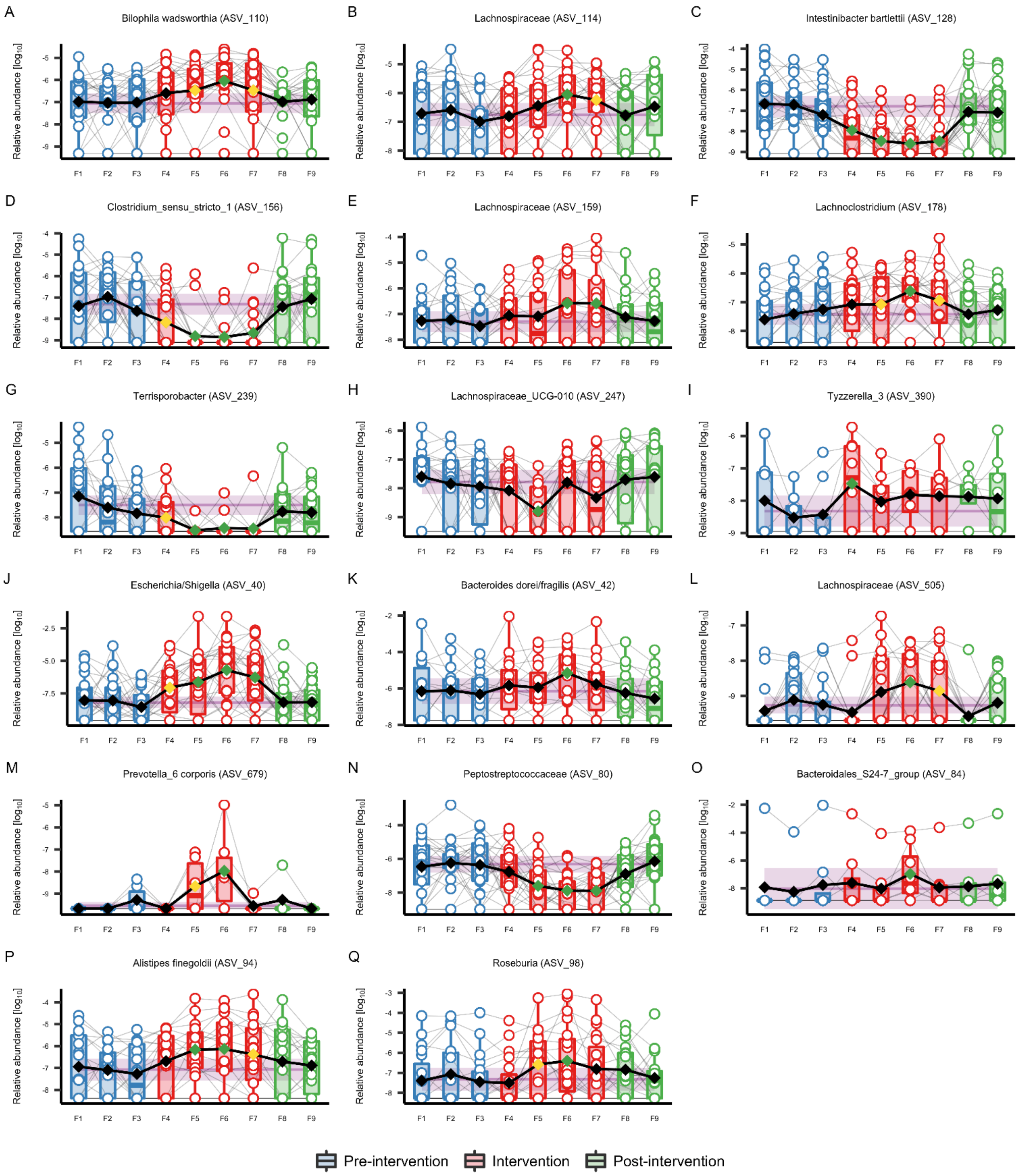

ESM Figure 1.

Metformin responsive amplicon sequence variants.

Bacterial amplicon sequence variants (ASV) exhibiting a change in relative abundance during the metformin intervention. Boxes represent interquartile range (IQR), with the inside line representing the median. Whiskers represent values within 1.5×IQR of the first and third quartiles. Circles represent individual samples with lines connecting samples from the same individual. The purple band represents the pre-intervention mean and 95% confidence limits averaged across the three pre-intervention time points. Diamonds and connecting lines represent mean values, with yellow and green diamonds respectively representing nominal ($p<0.05$) and false discovery rate adjusted ($q<0.05$) significant differences from the averaged pre-intervention mean. The relative abundance at each time point during the intervention was compared to the averaged pre-intervention mean by linear mixed model regression analysis of variance. Only ASVs with a significant change at least one time point following correction for false discovery rate is presented.

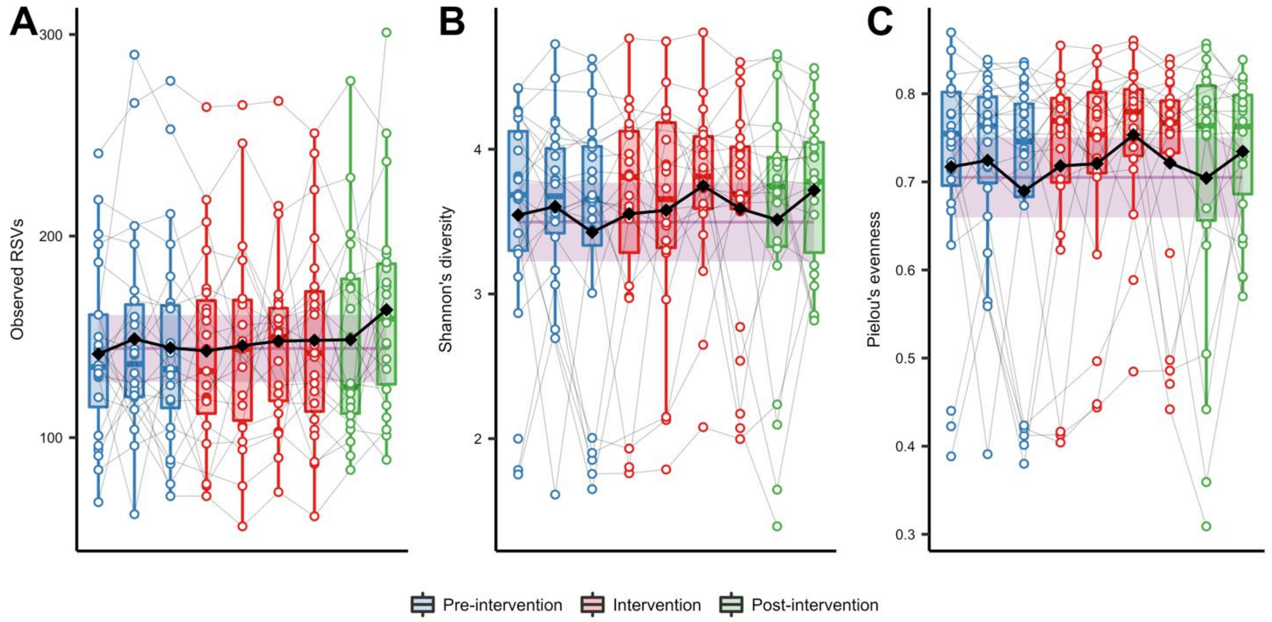

ESM Figure 2.

Effect of metformin on gut microbiota diversity. Change in microbial richness defined as the number of unique amplicon sequence variants (ASV) [A], overall diversity represented by Shannon's entropy [B] and evenness represented by Pielou's index [C] during and after the metformin intervention. Samples were rarefied to an equal sequencing depth of 10,000 reads. Boxes represent interquartile range (IQR), with the inside line representing the median. Whiskers represent values within 1.5×IQR of the first and third quartiles. Circles represent individual samples with lines connecting samples from the same individual. Diamonds and connecting lines represent mean values. The purple band represents the pre-intervention mean and 95% confidence limits averaged across the three pre-intervention time points.

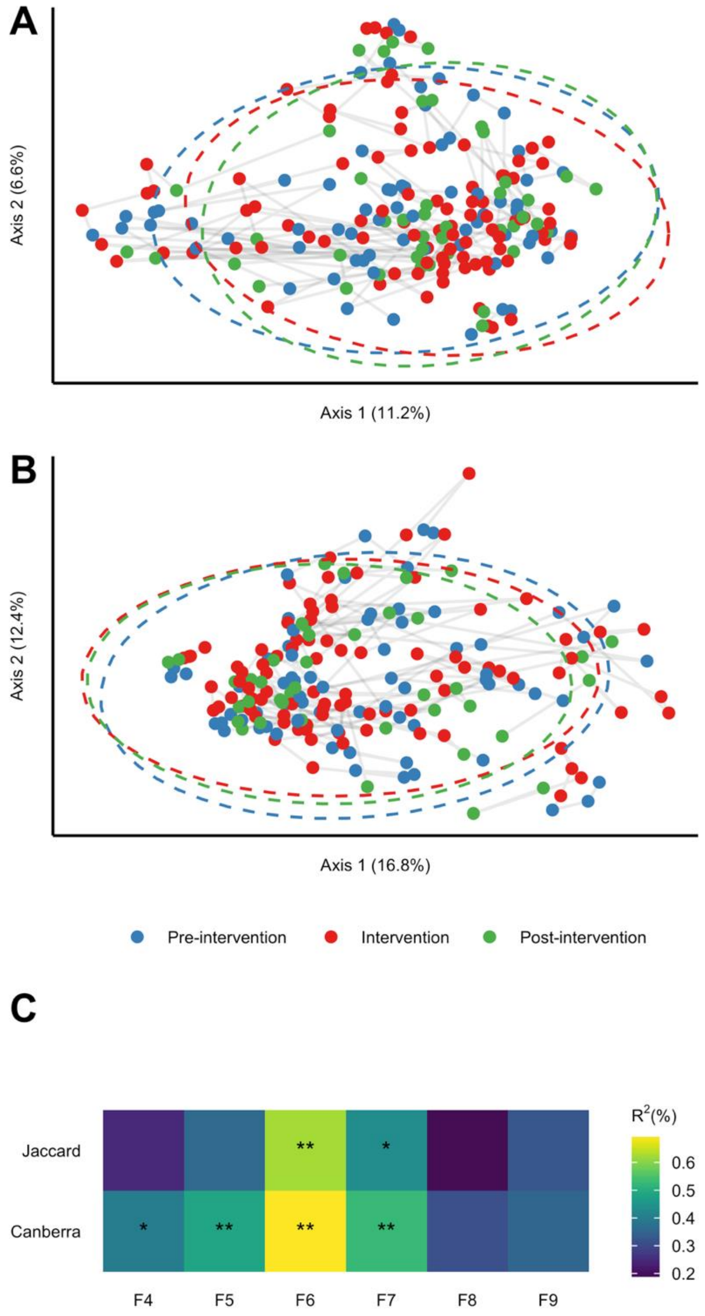

ESM Figure 3

Effect of metformin on gut bacterial community structure and membership
[A] Unconstrained principal coordinate analysis of gut microbial community structure based on Canberra distances and [B] community membership based on Jaccard distances. Points are individual samples projected on the 1st and 2nd principal coordinate axes with lines connecting consecutive samples from the same individual and ellipses representing the 95% confidence intervals of a multivariate normal distribution stratified by study period. [C]

Heatmap showing variation in Jaccard and Canberra distances explained (R2) by the contrast between pre-intervention samples and samples collected during (F4-F7) and after (F8-F9) the metformin intervention as determined by permutational multivariate analyses of variance (PERMANOVA). * p<0.05, ** p<0.01. Samples were rarefied to an equal sequencing depth of 10,000 reads prior to ordination and PERMANOVA analyses.
